# What Are the Optimal Levels of Time Perspectives? Deviation from the Balanced Time Perspective-Revisited (DBTP-r)

**DOI:** 10.5334/pb.487

**Published:** 2020-06-25

**Authors:** Konrad S. Jankowski, Marcin Zajenkowski, Maciej Stolarski

**Affiliations:** 1 Department of Psychometrics and Psychological Diagnosis, Faculty of Psychology, University of Warsaw, Warsaw, PL

**Keywords:** Zimbardo Time Perspective Inventory, Time perspective, Balanced time perspective, Well-being, Affect, Life satisfaction

## Abstract

Balanced time perspective (BTP) describes a tendency to focus on past, present and future time horizons that fosters well-being and positive life outcomes. Deviation from the balanced time perspective is a widespread method to measure the balance, but it makes assumptions regarding levels of time perspectives constituting BTP. In the present research we aimed to test the assumptions regarding levels of time perspectives constituting BTP by testing associations between time perspectives and domains of well-being in four independent samples (*N* = 1150). The results showed that higher well-being was fostered by greater past positive (PP) and future (F) and lower past negative (PN) and present fatalistic (PF) time perspectives in a linear manner. As for the present hedonistic (PH) perspective, the results were inconsistent indicating that this time orientation can be unrelated to well-being or related in an inverse U-shape manner. In the light of our results the optimal values for the deviation from the balanced time perspective, as measured with the Zimbardo Time Perspective Inventory, should be revisited and changed into PN 1, PP 5, PF 1, PH 3.4, F 5, with careful consideration whether or not to incorporate PH into the formula for the deviation from the balanced time perspective at all. We also showed that the deviation from the balanced time perspective using the above values better predicts well-being than the one using previously assumed levels.

## Introduction

Time perspectives describe individuals’ views on the past, present, and future, which are relatively stable individual characteristics. Zimbardo and Boyd (1999) distinguished five time perspectives that had emerged in empirical studies and were measured by the most frequently used questionnaire in the field, the Zimbardo Time Perspective Inventory. These perspectives were: Past-Negative (PN), a tendency to recall bad memories evoking negative affect; Past-Positive (PP), a tendency to recall good memories evoking positive affect; Present-Hedonistic (PH), a tendency to behave under the influence of appetitive stimuli; Present-Fatalistic (PF), faith in destiny and lack of control over one’s life; and Future (F), a focus on future accompanied by a tendency to organise behaviour to achieve planned goals.

Recent studies have focused on the importance of the balanced time perspective (BTP), reflecting a harmony across different time orientations (Boniwell, Osin, Linley, & Ivanchenko, 2010; Drake, Duncan, Sutherland, Abernethy, & Henry, 2008). Individuals with BTP have exhibited greater levels of subjective well-being compared with those who were less balanced (Boniwell et al., 2010; Zhang, Howell, & Stolarski, 2013). What is more, BTP predicts well-being over and above its two most powerful personality-level predictors – extraversion and neuroticism (Stolarski, 2016). Specifically, the studies have shown that in individuals with high levels of temporal balance, the effects of these personality traits on well-being were no longer significant, providing evidence for the moderating-attenuating role of BTP on the relationship between personality and well-being.

Researchers have proposed three methods to assess BTP based on the Zimbardo Time Perspective Inventory: a cut-off scores method (Drake et al., 2008); cluster analysis (Boniwell et al., 2010); and deviation from the balanced time perspective (DBTP; Stolarski, Bitner, & Zimbardo, 2011). In the comparative study of the three methods, Zhang et al. (2013) showed that the latter approach had the greatest predictive validity for subjective well-being. DBTP is calculated for every single individual and is defined as a Euclidean distance between optimal (o) and empirical (e) levels of time perspectives:

{\rm{DBTP}} = \sqrt {{{\left( {oPN - ePN} \right)}^2} + {{\left( {oPP - ePP} \right)}^2} + {{\left( {oPF - ePF} \right)}^2} + {{\left( {oPH - ePH} \right)}^2} + {{\left( {oF - eF} \right)}^2}}

The empirical levels are scores obtained by an individual in the Zimbardo Time Perspective Inventory (Zimbardo & Boyd, 1999). The optimal levels of time perspectives are the same for every individual and chosen arbitrary. Specifically, Stolarski et al. (2011) stated that ‘following Zimbardo and Boyd’s (2012) proposal (cf. www.thetimeparadox.com/surveys), and based on Zimbardo and Boyd’s (2012) collective cross-cultural database, we defined a “high” score on past positive as 4.60, a “moderately high” score on present hedonism and future as 3.90 and 4.00 respectively, and “low” on past negative and present fatalism as 1.95 and 1.50 respectively’. The values indicating optimal levels of time perspectives corresponded to percentile distribution of scores from ongoing studies running on the above-mentioned website. Specifically, they represented low scores on PN (corresponding to 10%), high scores on PP (90%), low scores on PF (10%), and moderately high on PH and F (80%). Percentiles defining ‘high’, ‘moderately high’, or ‘low’ values defining optimal levels of time perspectives were arbitrarily chosen, making the optimal values arbitrary as well. Specifically, Zimbardo and Boyd (2012) stated, ‘the red dots and lines [reflecting ideal profile] are not associated with the data in any way. It is simply our idea of what an ideal time perspective looks like’. What is more, the values corresponding to the above-mentioned percentiles drifted when the database was updated on 17 September 2012, and now they are PN 2.1, PP 3.67, PF 1.67, PH 4.33, and F 3.69. The characteristics and sizes of the previous samples are not known.

The use of the above optimal values imposes quadratic associations between time perspectives and indicators of optimality (e.g., well-being), because individuals can score above (maximum = 5) or below (minimum = 1) the optimal values (Zimbardo & Boyd, 1999). However, the previous studies have not reported tests of quadratic associations between time perspectives and indicators of optimality. The previous studies (Boniwell et al., 2010; Desmyter & De Raedt, 2012; Drake et al., 2008; Sailer et al., 2014; Sobol-Kwapińska & Jankowski, 2016; Stolarski, Matthews, Postek, Zimbardo, & Bitner, 2014; Zhang et al., 2013) tested linear associations and suggested that optimal values should be either 1 (PN, PF) or 5 (PP, PH, F), as these are the extreme scores from the Zimbardo Time Perspective Inventory (Zimbardo & Boyd, 1999). Concerns regarding the optimal values have been also raised by other researchers (McKay et al., 2018).

In the present paper, for the first time the optimal values will be determined through empirical tests indicating what levels of time perspectives are optimal for well-being, i.e., by considering both linear and quadratic models to check whether non-extreme values (e.g., ‘moderately high’) can indeed be the most optimal. After Boniwell and Zimbardo (2004) and Zhang et al. (2013) we defined optimal levels of time perspectives as those maximising subjective well-being. Based on conceptualizations of subjective well-being (Sumner, 1996) we will assess both cognitive (life satisfaction) and affective facets (positive affect, negative affect, energetic arousal, tense arousal, hedonic tone, depression symptoms). Specifically, we will predict each facet of well-being by each time perspective using linear versus quadratic regression models. As opposed to the original research (Zimbardo & Boyd, 2012) our tests will be based on samples of known demographic characteristics.

## Material and Methods

### Participants

In the current investigation we analysed data from four independent samples. Sample 1 consisted of 232 participants (123 female and 109 male) with a mean age of 23.62 (SD = 3.80) ranging from 18 to 39; in sample 2 there were 219 subjects (160 female and 59 male) and their mean age was 21.22 (SD = 2.51; range 18–40); in sample 3 there were 276 subjects (137 female, 139 male) with a mean age of 25.13 (SD = 2.65; range: 18–49); and sample 4 consisted of 423 participants (217 female and 206 male) and their mean average age was 22.77 (SD = 3.53; range: 18–40).

In samples 1, 3, and 4, volunteer participants were recruited via publicly accessible social networking websites, and all volunteering adults were invited to take part in the studies. Participants completed a packet containing a variety of self-report questionnaires and laboratory tasks. Each participant was tested in the laboratory at the Faculty of Psychology, University of Warsaw, and was offered a small gift (worth approximately 10 USD) for taking part in the study. Sample 2 was recruited just before classes began at university, where participants were approached by pollsters. They volunteered in the study without remuneration. This study, including the consent process, was approved by the ethics committee of the Faculty of Psychology, University of Warsaw. Verbal informed consent with an information sheet was obtained from all participants, in order to assure complete anonymity. Participation was voluntary and participants were allowed to reject or withdraw at any point with no disadvantage to their treatment. The data of the study are available on a public repository (https://osf.io/5qe4d/).

### Measures

#### Time Perspectives

*Time perspectives* were measured using the Zimbardo Time Perspective Inventory (ZTPI; Zimbardo & Boyd, 1999) in the Polish translation by Kozak and Mażewski (2007). The questionnaire comprises 56 items rated on a five-point, Likert-type scale. It has five scales, namely: Past-Positive, Past-Negative, Present-Fatalistic, Present-Hedonistic, and Future, with sufficient internal consistencies shown in previous research (Cronbach alphas ranging between .74 and .82) and the current study (Table 1).

**Table 1:** Descriptive statistics of all variables from four samples.

	*M*	*SD*	Min	Max	α

**Sample 1 (*N* = 232)**					

Past Negative	2.92	.76	1.00	4.70	.84
Past Positive	3.40	.60	1.00	4.78	.66
Present Hedonism	3.31	.56	1.53	4.80	.81
Present Fatalism	2.43	.68	1.11	4.78	.78
Future	3.56	.60	2.00	4.92	.79
Life Satisfaction	22.60	5.87	5.00	35	.85
**Sample 2 (*N* = 219)**					

Past Negative	3.01	.77	1.00	4.80	.83
Past Positive	3.43	.53	1.44	4.56	.54
Present Hedonism	3.40	.60	1.00	4.60	.79
Present Fatalism	2.52	.60	1.00	4.11	.67
Future	3.50	.64	1.00	4.77	.80
Life Satisfaction	21.97	5.52	8.00	35.00	.80
Depression	17.60	10.44	1.00	47.00	.90
**Sample 3 (*N* = 276)**					

Past Negative	3.10	.88	1.00	5.00	.86
Past Positive	3.47	.77	1.44	5.00	.81
Present Hedonism	3.40	.66	1.00	5.00	.86
Present Fatalism	2.48	.67	1.00	5.00	.73
Future	3.43	.64	1.38	4.92	.81
Positive Affect	32.48	7.35	10.00	50.00	.87
Negative Affect	18.37	7.84	10.00	50.00	.90
**Sample 4 (*N* = 423)**					

Past Negative	2.98	.77	1.20	4.80	.83
Past Positive	3.48	.65	1.56	4.89	.71
Present Hedonism	3.47	.57	1.73	4.93	.82
Present Fatalism	2.60	.611	1.22	4.11	.69
Future	3.42	.62	1.15	4.85	.81
Energetic Arousal	22.13	4.66	10.00	32.00	.84
Tense Arousal	16.86	4.10	8.00	30.00	.78
Hedonic Tone	22.99	4.92	10.00	32.00	.91

#### Well-Being

*Life satisfaction* was measured using Satisfaction With Life Scale (SWLS; Diener, Emmons, Larsen, & Griffin, 1985) in the Polish adaptation by Jankowski (2015). The scale comprises five items measuring one’s cognitive judgements of general satisfaction with their life, which are rated on a seven-point, Likert-type scale. Previous study showed its high test-retest reliability (.85–.93 depending on tested time period) and internal consistency (Cronbach alpha amounts to .86). Internal consistency in the current study was also high (Table 1)

*Positive and negative affect* was measured with the Positive And Negative Affect Schedule (PANAS; Watson, Clark, & Tellegen, 1988), one of the most widely used scales measuring positive and negative affectivity. It may be used to measure affect in a variety of contexts, including both state affect and stable dispositional tendency to experience positive and negative emotions (trait affect). In the present study, the Polish adaptation of PANAS was used (SUPIN C20; Brzozowski, 2010). The C20 version of the questionnaire measures trait affect and comprises 20 items, grouped into two scales – Positive Affect and Negative Affect. Internal consistencies in Polish validation studies ranged between .73 and .95 and they were also acceptable in the current research (Table 1).

*Mood* was assessed using the Polish translation of *UWIST Mood Adjective Check List* (UMACL; Matthews, Jones, & Chamberlain, 1990). Participants were presented with a list of 24 adjectives and a four-point, Likert-type response format (ranging from 1 = *strongly disagree* to 4 = *strongly agree*) to describe their present mood state. The scale is divided into three subscales, each consisting of eight items: energetic arousal (with poles: energetic-tired); tense arousal (nervous-relaxed); and hedonic tone (pleasant-unpleasant). Internal consistency in the current research was high (Table 1).

*Depression symptoms* were measured with the Center for Epidemiological Studies – Depression scale (CES-D; Radloff, 1977) – one of the most widely used screeners for depressive symptoms in non-clinical samples. The scale is composed of 20 items asking about the occurrence of depression symptoms within the previous week. The symptoms belong to different domains, i.e., somatic, depressed, and positive affect, and interpersonal relations; higher scores indicate greater depressiveness. The Polish adaptation (Jankowski, 2017) proved good psychometric properties, e.g., internal consistency was .90. Internal consistency was also high in the current research (Table 1).

### Analyses

At first, descriptive statistics (*M, SD*, min, max, and Cronbach’s alpha) were calculated. To test linear vs quadratic associations between TPs and indicators of well-being, linear regressions were used. For regression analyses, all scores were transformed into *z*-scores. In all analyses, each time perspective was entered as a predictor in step 1 (linear trend), followed by the squared time perspective score entered in step 2 (quadratic trend), whereas each indicator of well-being was entered as a dependent variable.

## Results

In Table 1, descriptive statistics and reliabilities for all measures from four samples are presented, showing that all measures have acceptable internal consistency (with the exception for PP displaying alpha below .70 in two samples) and vary across full spectrum. Tables 2, 3, 4 show results of regression models conducted separately for each time perspective and particular well-being dimension.

**Table 2:** Results of regression analyses: each time perspective was entered as predictor in step 1, followed by the squared time perspective score entered in step 2 and well-being indicators (Life satisfaction and Depression) as dependent variables.

		Sample 1 (*N* = 232)				Sample 2 (*N* = 219)							

		Life satisfaction				Life satisfaction				Depression			

Step		*B*	β	*R*^2^ adj	Δ*R*^2^	*B*	β	*R*^2^ adj	Δ*R*^2^	*B*	β	*R*^2^ adj	Δ*R*^2^

1	Intercept	22.60		.261	.264**	21.97		.235	.238**	17.61		.239	.243**
	Past Negative	–3.98	–.51**			–3.51	–.49**			6.71	.49**		
2	Intercept	22.62		.257	.000	22.17		.234	.002	17.26		.238	.002
	Past Negative	–3.98	–.51**			–3.55	–.49**			6.78	.50**		
	Past Negative squared	–.03	–.01			–.34	–.05			.60	.05		
1	Intercept	22.84		.004	.008	21.97		.023	.028*	17.61		.042	.042*
	Past Positive	1.09	.09			1.70	.17*			–4.00	–.21**		
2	Intercept	22.71		.002	.002	22.13		.020	.002	17.74		.043	.000
	Past Positive	1.47	.12			1.54	.15*			–4.14	–.21**		
	Past Positive squared	.73	.06			–.55	–.04			–.48	–.02		
1	Intercept	22.60		.000	.000	21.97		.000	.000	17.61		.011	.015
	Present Hedonism	–.12	–.01			.20	.02			2.16	.12		
2	Intercept	22.56		–.001	.000	22.05		.000	.001		17.40	.007	.001
	Present Hedonism	–.11	–.01			.13	.01			2.34	.13		
	Present Hedonism squared	.13	.01			–.24	–.03			.60	.04		
1	Intercept	22.60		.071	.075**	21.97		.000	.005	17.61		.030	.035*
	Present Fatalism	–2.38	–.27**			–.63	–.07	.000	.000	3.24	.17*		
2	Intercept	22.00		.086	.019*	21.99				17.49		.026	.000
	Present Fatalism	–2.84	–.33**			–.63	–.07			3.25	.19*		
	Present Fatalism squared	1.32	.15*			.21	.02			.32	.01		
1	Intercept	22.87		.022	.026*	21.97		.016	.020*	17.61		.004	.009
	Future	2.05	.16*			1.22	.14			–1.52	–.09		
2	Intercept	22.65		.021	.004		22.02		.011	.000	17.98	.002	.003
	Future	2.27	.18*			1.12	.14			–1.73	–.11		
	Future squared	1.10	.06			–.11	–.01			–.91	–.06		

* *p* < .05; ** *p* < .01.

**Table 3:** Results of regression analyses: each time perspective was entered as predictor in step 1, followed by the squared time perspective score entered in step 2 and well-being indicators (Positive affect and Negative affect) as dependent variables.

		Sample 3 (*N* = 276)							

		Positive affect				Negative affect			

Step		*B*	β	*R*^2^ adj	Δ*R*^2^	*B*	β	*R*^2^ adj	Δ*R*^2^

1	Intercept	32.48		.005	.008	18.37		.138	.141**
	Past Negative	–.76	–.09			3.35	.37**		
2	Intercept	32.68		.002	.001	17.03		.172	.037**
	Past Negative	–.79	–.10			3.56	.40**		
	Past Negative squared	–.25	–.03			1.74	.20**		
1	Intercept	32.48		.054	.057**	18.37		.025	.025*
	Past Positive	2.27	.24**			–1.59	–.16*		
2	Intercept	32.66		.051	.001	18.23		.025	.000
	Past Positive	2.18	.23**			–1.52	–.15		
	Past Positive squared	–.29	–.03			.24	.02		
1	Intercept	32.48		.080	.083**	18.37		.026	.029*
	Present Hedonism	3.21	.29**			2.02	.17*		
2	Intercept	32.49		.077	.000	18.10		.025	.003
	Present Hedonism	3.20	.29**			2.11	.18*		
	Present Hedonism squared	–.03	.00			.62	.06		
1	Intercept	32.48		.000	.001	18.37		.143	.146**
	Present Fatalism	.22	.02			4.46	.38**		
2	Intercept	32.61		.000	.000	17.27		.181	.040**
	Present Fatalism	.27	.03			4.07	.35**		
	Present Fatalism squared	–.28	–.03			2.43	.20**		
1	Intercept	32.48		.027	.030**	18.37		.004	.008
	Future	1.98	.17*			–1.06	–.09		
2	Intercept	32.25		.025	.002	18.83		.007	.006
	Future	2.13	.19*			–1.36	–.11		
	Future squared	.558	.04			–1.13	–.08		

* *p* < .05; ** *p* < .01.

**Table 4:** Results of regression analyses: each time perspective was entered as predictor in step 1, followed by the squared time perspective score entered in step 2 and well-being indicators (Energetic arousal, Tense arousal and Hedonic tone) as dependent variables.

		Sample 4 (*N* = 423)											

		Energetic arousal				Tense arousal				Hedonic tone			

Step		*B*	β	*R*^2^ adj	Δ*R*^2^	*B*	β	*R*^2^ adj	Δ*R*^2^	*B*	β	*R*^2^ adj	Δ*R*^2^

1	Intercept	22.15		.123	.126**	16.85		.055	.057**	23.00		.161	.164**
	Past Negative	–2.15	–.35**			1.27	.24**			–2.59	–.40**		
2	Intercept	22.41		.126	.004	16.69		.055	.002	23.10		.160	.001
	Past Negative	–2.13	–.35**			1.26	.24**			–2.59	–.40**		
	Past Negative squared	–.44	–.07			.27	.05			–.17	–.02		
1	Intercept	22.14		.017	.019*	16.86		.012	.014	23.00		.043	.045**
	Past Positive	1.01	.14*			–.74	–.12			1.59	.21**		
2	Intercept	22.18		.015	.000	16.86		.009	.000	23.05		.041	.000
	Past Positive	.99	.14*			–.74	–.12			1.59	.21**		
	Past Positive squared	–.11	–.01			.03	.00			–.14	–.02		
1	Intercept	22.14		.000	.000	16.86		.000	.002	23.00		.000	.000
	Present Hedonism	.16	.02			.30	.04			–.058	–.01		
2	Intercept	22.27		.000	.001	16.91		.000	.000	23.00		.000	.000
	Present Hedonism	.14	.02			.30	.04			–.062	–.01		
	Present Hedonism squared	–.40	–.04			–.17	–.02			–.11	–.01		
1	Intercept	22.15		.049	.051**	16.85		.055	.057**	23.00		.068	.070**
	Present Fatalism	–1.74	–.23**			1.60	.24**			–2.16	–.27**		
2	Intercept	22.30		.048	.002	16.60		.058	.005	23.19		.068	.002
	Present Fatalism	–1.73	–.23**			1.60	.24**			–2.53	–.26**		
	Present Fatalism squared	–.43	–.04			.69	.07			–.53	–.05		
1	Intercept	22.13		.076	.078**	16.86		.004	.006	23.00		.041	.043**
	Future	2.09	.28**			–.53	–.08			1.65	.21**		
2	Intercept	22.27		.075	.002	16.86		.002	.000	22.77		.042	.003
	Future	201	.27**			–.53	–.08			1.78	.22**		
	Future squared	–.37	–.04			.00	.00			.53	.06		

* *p* < .05; ** *p* < .01.

PN was linearly related to indicators of well-being in eight out of nine models, where it explained from 5.7% (tense arousal) to 26.4% (life satisfaction) of variance, and in the eight models greater PN was linked to lower well-being (Tables 2, 3 and 4). The model predicting positive affect based on PN was nonsignificant (Table 3). At the same time, quadratic trends were nonsignificant in eight models, while the quadratic model predicting negative affect was statistically significant (Table 3). Specifically, a U-shaped association appeared with the minimum situated below the mean PN value, indicating that the optimal level of PN minimising negative affect would be below the PN mean, while both greater and lower values of PN would increase negative affect. However, given that the quadratic association appeared for only this one well-being domain, and it explained only slightly more variance than the linear trend (17.8% vs 14.1%, respectively), we cannot conclude that the association between PN and well-being is generally quadratic. On the contrary, these results indicate that PN predicts well-being in a linear manner – this explanation is also in line with the principle of parsimony.

PP was related to indicators of well-being in seven out of nine linear models, which explained from 1.9% (energetic arousal; Table 4) to 5.7% (positive affect; Table 3) of variance; in these models greater PP was linked to higher well-being. Linear models predicting life satisfaction in sample 1 and tense arousal based on PP were nonsignificant. At the same time, quadratic trends were nonsignificant in all nine models.

PH was related to only two indicators of well-being out of nine linear models tested. Specifically, greater PH fostered more positive affect (8.3% of variance) and more negative affect (2.5% of variance; Table 3), while quadratic associations were nonsignificant. This result seems ambiguous as it means that PH can play both in favor and against well-being at the same time.

PF was linearly related to indicators of well-being in seven out of nine models, which explained from 3.5% (depression; Table 2) to 14.6% (negative affect; Table 3) of variance and PF predicted lower levels of well-being in these models. The model predicting positive affect based on PF, as well as the model predicting life satisfaction in sample 2, was nonsignificant. There appeared to be two quadratic trends: PF predicted life satisfaction and negative affect in a U-shaped manner, with the optimal levels of PF above its mean (Table 2) and below its mean (Table 3), respectively. Again, given that the quadratic association appeared in only two models, explaining only slightly more variance than the linear models (life satisfaction 9.4% vs 7.5%, respectively; negative affect 15.0% vs 14.6%, respectively), we cannot conclude that the association between PF and well-being is the quadratic one at large. On the contrary, the results suggest that PF predicts well-being rather in a linear manner, which is also more parsimonious than the quadratic trend.

F was related to indicators of well-being in six out of nine linear models, which explained from 2.0% (life satisfaction; Table 2) to 7.8% (energetic arousal; Table 4) of variance, and in these models greater F was linked to higher well-being. Three linear models, predicting depression, negative affect, and tense arousal based on F were nonsignificant. At the same time, quadratic trends were nonsignificant in all nine models.

Summing up, the regression results show that linear associations between TPs and well-being are widespread, given that among nine models they appeared in eight models with PN, seven models with PP or PF, and six models with F, but only in two models with PH. Considering direction of linear associations, higher levels of PP and F and lower levels of PN and PF were associated with higher well-being. Ambiguous association appeared in the case of PH, as the results show that in sample 3 (no associations in other samples) higher levels of this TP are linked to high levels of both positive and negative affect.

As for the quadratic trend, it was irrelevant in case of all TPs except PF – the squared score of PF was statistically significant in sample 1, where it accounted for an additional 1.9% of the variance in life satisfaction (over 7.4% explained by the linear trend; Table 2) and in sample 3, where it explained an additional 4% of the variance in negative affect (over 14.6% explained by the linear trend; Table 3). Furthermore, the PN squared score was statistically significant in sample 3, where it accounted for an additional 3.7% of the variance in negative affect (over 14.1% explained by the linear trend; Table 3).

## Discussion

In the present paper we challenged the idea of the time perspective optimal values indicated by Zimbardo and Boyd (2012) and further developed by Stolarski et al. (2011) in the DBTP formula. Based on four independent samples, testing nine models with seven indicators of well-being, we show that there is little evidence that the currently used optimal values are valid, as quadratic trends imposed by them are nonexistent. This is the first study searching for quadratic associations between TPs and external outcomes. Below, we discuss results regarding each time perspective, propose empirically based optimal values, and apply them to DBTP. We show that DBTP with the novel values better predict well-being than DBTP using the previous values.

### Past-Negative

The linear associations between PN and well-being found in our studies fit previous reports, whereas quadratic models have not been reported until now. For instance, Boniwell et al. (2010) showed in British and Russian samples that greater PN was linearly related to lower well-being in such domains as positive and negative affect, actualisation of potential, life satisfaction, happiness, purpose in life, self-efficacy, and optimism. Drake et al. (2008) showed in a British sample that greater PN was linearly related to lower happiness. Similarly, Zhang et al. (2013), in four American samples, revealed that greater PN was linearly related to lower well-being in domains of positive and negative affect, life satisfaction, and happiness. Also, Sobol-Kwapińska and Jankowski (2016) showed more pronounced PN linearly related to lower self-esteem, life satisfaction, and optimism in a Polish sample; Stolarski et al. (2014) showed that higher PN was linearly related to disadvantageous affectivity denoted by lower energetic arousal and hedonic tone, and higher tense arousal. Furthermore, Zimbardo and Boyd (1999) showed that higher PN was linked to more depression symptoms. Overall, our results, together with those from previous studies, indicate that linear negative association between PN and well-being is justified; therefore, the minimum value (1) of this TP seems to be most plausibly the optimal one.

### Past-Positive

Results showing that greater PP is linearly related to higher well-being are in line with previous reports showing linear associations between PP and indicators of well-being. For instance, Boniwell et al. (2010) showed in two samples that greater PP was linearly related to higher well-being in domains of positive and negative affect, actualisation of potential, life satisfaction, happiness, purpose in life, and optimism, but was unrelated to self-efficacy. Greater PP was linearly related to higher well-being in terms of happiness (Drake et al. 2008; Zhang et al., 2013), life satisfaction, and positive and negative affect (Zhang et al., 2013). Also, Sobol-Kwapińska and Jankowski (2016) showed more pronounced PP was linearly related to higher self-esteem, life satisfaction, and optimism. Stolarski et al. (2014) showed that higher PP was linearly related to advantageous affectivity (higher energetic arousal and hedonic tone, lower tense arousal). Additionally, Zimbardo and Boyd (1999) showed that higher PP was linked to fewer depression symptoms. Overall, our results, together with those from previous studies, indicate that the linear positive association between PP and well-being is justified and, therefore, the maximum value (5) of this TP seems to be the most plausible as the optimal one.

### Present-Hedonistic

Results regarding PH were ambiguous. Also, when looking at previous studies, it appears that associations of PH with well-being domains are inconsistent. For instance, in the study by Boniwell et al. (2010), higher PH was related to more positive affect, but unrelated to negative affect, whereas a few different aspects of well-being (actualisation of potential, happiness, optimism) were positively related to PH. At the same time, life satisfaction and purpose in life were unrelated to PH. Contrary to these findings, Drake et al. (2008) showed that more pronounced PH lowers happiness. In the Zhang et al.’s (2013) research, however, PH was related positively, but weakly, to life satisfaction in three samples (no association in their fourth sample), and positively to happiness and positive affect, while association with negative affect did not appear in all samples. Finally, in the study by Sobol-Kwapińska and Jankowski (2016), PH was unrelated to all indicators of well-being (self-esteem, life satisfaction, optimism), whereas the Zimbardo and Boyd (1999) study showed greater PH correlated with more symptoms of depression.

The issue of adaptiveness of the PH dimension seems even more complicated if we look at its nomological network (see Stolarski, Fieulaine, & van Beek, 2015). The dimension proved to be associated with many prerequisites or correlates of well-being, but both in positive and negative directions. On the one hand, the list of PH correlates includes such adaptive features as curiosity (Kashdan, Rose, & Fincham, 2004), large social network with more support (Holman & Zimbardo, 2009), or more frequent physical activity (Daugherty & Brase, 2010). On the other hand, PH proved associated with greater consumption of psychostimulants and addiction (Daugherty & Brase, 2010; Keough, Zimbardo & Boyd, 1999), mania susceptibility (Gruber, Cunningham, Kirkland, & Hay, 2012), or pathological gambling (MacKillop, Anderson, Castelda, Mattson, & Donovick, 2006). This suggests that in cases of PH, using its extreme values in the DBTP equation may be not a reasonable solution.

The presented results indicate ambiguous outcomes regarding the nature of association between PH and well-being. Although quadratic association did not appear in our data considering a single well-being domain, there might be arguments to support an inverse U-shaped relationship when different domains are considered – i.e., positive affect and negative affect. To reveal the optimal value of PH maximising well-being in terms of positive affect and negative affect, one may find an intersection point of the two lines describing the two associations: PH – positive affect (standardised positive affect = –10.93 + 3.21*PH) vs PH – inversed negative affect (standardised inversed negative affect = 6.90 – 2.02*PH), which appears for 3.4 scores in PH. Nevertheless, given the ambiguous results also found in previous studies, it can be questioned whether an optimal value of PH exists at all.

Taking into account the nature of PH, it seems possible that PH is rather associated with hedonic than eudaimonic (see Tiberius & Hall, 2010) aspects of well-being. However, in a study including both hedonic and eudaimonic aspects of well-being reported by Zhang et al. (2013; study 3), PH displayed only slightly stronger association with typically hedonic Positive Affect (*r* = .26) than with gratitude (*r* = .19), which is the core feature of eudaimonia.

### Present-Fatalistic

The linear associations found in our studies correspond to previous reports. For example, Boniwell et al. (2010) showed that greater PF was linearly related to lower well-being in all domains they studied. Similarly, Zhang et al.’s (2013) four samples revealed that greater PF was linearly related to lower well-being in all studied domains (weaker associations with positive affect). Similar results were obtained by Sobol-Kwapińska and Jankowski (2016) as well as by Stolarski et al. (2014), regarding all indicators of well-being they analysed. In line with these findings, Zimbardo and Boyd (1999) showed that higher PF was related to greater depressiveness. The only inconsistent result has been reported by Drake et al. (2008), who showed lack of association between PF and happiness. Overall, our results, together with those from previous studies, indicate that linear negative association between PF and well-being is justified, and, therefore, the minimum value (1) of this TP seems the most plausible as the optimal one.

### Future

Results on F are in line with previous reports showing linear associations between F and indicators of well-being, albeit, similarly to our findings, previous reports were less consistent regarding the predictive value of F for well-being. For instance, Boniwell et al. (2010) showed that greater F was linearly related to higher life satisfaction, purpose in life, and optimism, but unrelated to other domains of well-being (positive and negative affect, actualisation of potential, happiness, self-efficacy) in line with Drake et al. (2008), who did not observe associations of F with happiness. On the other hand, Zhang et al. (2013) showed that greater F consistently predicted higher life satisfaction, happiness, and positive affect across four samples, while associations with negative affect were less consistent. Also, Sobol-Kwapińska and Jankowski (2016) showed more pronounced F linearly related to higher self-esteem, life satisfaction, and optimism. Similarly, Stolarski et al. (2014) showed higher F linearly related to advantageous affectivity (higher energetic arousal and hedonic tone, lower tense arousal), and Zimbardo and Boyd (1999) showed that higher F was linked to fewer depression symptoms. Overall, our results, together with those from previous studies, indicate that the linear positive association between F and well-being is justified, and, therefore, the maximum value (5) of this TP seems the most plausible as the optimal one.

### Further directions

The presented study has several implications, but some limitations, as well. In light of our results, the optimal values for the DBTP should be revisited and changed into PN = 1, PP = 5, PF = 1, PH = 3.4, and F = 5 (values maximizing well-being), with careful consideration of whether to incorporate PH into the formula at all. The formula itself would then take the form of the Deviation from the Balanced Time Perspective – revisited (DBTP-r):

{\rm{DBTP - r}} = \sqrt {{{\left( {1 - ePN} \right)}^2} + {{\left( {5 - ePP} \right)}^2} + {{\left( {1 - ePF} \right)}^2} + {{\left( {3.4 - ePH} \right)}^2} + {{\left( {5 - eF} \right)}^2}}

Given the obtained results and arguments presented in the above sections of the discussion, we consider DBTP-r as a more valid indicator of BTP and advise using it in future studies on the construct. Comparison of predictive power of DBTP versus DBTP-r for well-being indicates that DBTP-r indeed performs slightly better than DBTP (Table 5); amongst eight correlations, DBTP-r, as compared to DBTP, was more strongly related to well-being in case of three indicators, similarly for four indicators, and less strongly for one indicator (Table 5). What is more, if we abandon incorporating PH to the formula, the optimal values could be simply summed up (PN and PP after inversion) to create a score representing BTP.

**Table 5:** Pearson correlations between indicators of well-being and deviation from the balanced time perspective in original (DBTP) and revisited (DBTP-r) form, and comparison (z) of correlation coefficients for DBTP and DBTP-r.

	Sample 1 (*N* = 232)	Sample 2 (*N* = 219)		Sample 3 (*N* = 276)		Sample 4 (*N* = 423)		

	Life satisfaction	Life satisfaction	Depression	Positive affect	Negative affect	Energetic arousal	Tense arousal	Hedonic tone

DBTP	–.461***	–.376***	.318***	–.236***	.407***	–.388***	.267***	–.415***
DBTP-r	–.540***	–.418***	.383***	–.203***	.431***	–.402***	.272***	–.434***
*z*	4.267***	1.948*	2.908**	–1.715*	1.343	1.055	.359	1.453

* *p* < .05; ** *p* < .01; *** *p* < .001; *z* = test statistic for comparison of correlations from dependent samples.

It should be noted that our recommendation is not the only proposed alteration of the classical DBTP formula. Recently, a revised DBTP version was proposed, taking into account the distinction between Future-Positive and Future-Negative (Rönnlund, Åström, & Carelli, 2017). The DBTP-E (E for Extended) accepts the ‘optimal’ points applied in the original DBTP, adding a novel component to the DBTP equation, indicating the discrepancy between optimal and empirical levels of Future-Negative. The authors calculated the optimal Future-Negative point per analogiam to Past-Negative (10^th^ percentile). Our consideration did not refer to the DBTP-E as data used for the present analyses were collected using the 56-item version of the ZTPI, not the broadened, 64-item Swedish ZTPI. Therefore, future analyses should determine whether the extreme score (1) in the Future-Negative subscale would be more justified than the one proposed by Rönnlund and colleagues (1.8 points).

Future studies could also add to the DBTP-r’s validity by broadening its nomological network. In the present paper we focused on DBTP-r associations with various indicators of well-being, which was a natural choice taking into account both the initial conceptualisation of balanced time perspective (Zimbardo & Boyd, 1999) and the fact that the construct has been developed within the framework of positive psychology (Boniwell & Zimbardo, 2004; Zhang et al., 2013). The traditional DBTP coefficient proved to be associated with (for review see Stolarski, Zajenkowski, Jankowski, & Szymaniak, 2020): intelligence (Zajenkowski, Stolarski, Maciantowicz, Malesza, & Witowska, 2016), personality (Birkás, Matuz, & Csathó, 2018), self-compassion (Phillips, 2018), greater relationship satisfaction (Stolarski, Wojtkowska, & Kwiecińska, 2016), lower depression and anxiety (Papastamatelou, Unger, Giotakos, & Anthanasiadou, 2015), fewer PTSD symptoms after a traumatic experience (Stolarski & Cyniak-Cieciura, 2016), greater mindfulness (Stolarski, Vowinckel, Jankowski, & Zajenkowski, 2016), sense of coherence (Wiesmann, Ballas, & Hannich, 2018), greater self-control (Orkibi & Ronen, 2018), less compulsive buying (Unger, Lyu, & Zimbardo, 2018), less alcohol consumption (Loose et al., 2018), or more healthful leisure choices (Garcia & Ruíz, 2015). Determining whether the DBTP-r would prove more, or at least equally, predictive of these vital outcomes is an important task before the novel indicator becomes a commonly accepted way to assess temporal balance.

Another future research pathway is related to answering the question about cultural specificity vs generality of the DBTP-r. Time perspective is a phenomenon studied all around the world, and the ZTPI has been adapted and applied in a variety of nations and cultures. In some cultures, however (e.g., Japan), certain problems with the scale have been identified (see Sircova et al., 2014). In the present paper we present data collected solely in Poland. Thus, it seems important to test whether the DBPT-r remains a culture-free indicator of temporal balance.
